# Influence of Combined Pretreatments on Drying Time and Quality of Melon Chips Produced by Vacuum-Assisted Two-Way Infrared Drying

**DOI:** 10.17113/ftb.64.03.26.9444

**Published:** 2026-09-15

**Authors:** Gülsüm Çur, Serdar Uğurlu, Tahir Yücel, Emre Bakkalbaşı

**Affiliations:** 1 Department of Food Engineering, Faculty of Engineering, Van Yüzüncü Yıl University, Zeve Campus, 65080 Van, Türkiye; 2 Department of Plant and Animal Production, Yüksekova Vocational School, Hakkari University, Hakkari, 30100, Türkiye

**Keywords:** melon chips, pretreatments, quality properties, specific energy, vacuum-assisted infrared drying

## Abstract

**Research background:**

Infrared technology, which has become widespread in recent years due to its rapid drying effect, also causes some negative changes in products. To eliminate the adverse effects that occur during the drying of products, different pretreatments such as dipping, blanching, freezing and ultrasonic treatment are applied.

**Experimental approach:**

This study aims to determine the effect of different pretreatments (citric acid, ethanol, ultrasound, blanching and freezing) and their combinations on drying time and quality characteristics of melon chips produced by a novel vacuum-assisted two-way infrared dryer.

**Results and conclusions:**

The samples produced with the combination of citric acid/ethanol/ultrasound/freezing had the highest rehydration ratio (6.1 %), shortest drying time (72.5 min), lowest specific energy requirement (2.6 kWh/kg) and lowest shrinkage ratio (85.7 %). The best result in terms of colour values was obtained from the samples treated with the citric acid/ethanol combination. The 5-hydroxymethylfurfural (HMF) mass fractions on dry mass basis of melon chips produced with pretreatments including ultrasound were higher (74.9–118.0 mg/kg) than with other pretreatments. Multiple pretreatment applications caused a decrease in total phenolic content and antioxidant activity. The highest total phenolic content on dry mass basis, expressed as gallic acid equivalents (2565 mg/kg) and DPPH value, expressed as Trolox equivalents (7.5 mmol/g) were found in the samples immersed in citric acid. The most liked melon chips were the samples in which the citric acid/blanching combination was used.

**Novelty and scientific contribution:**

Infrared drying is emerging as an increasingly widespread drying technology. The primary innovation of this study is the investigation of how two-way vacuum-assisted infrared technology can mitigate or eliminate the disadvantages of infrared drying by leveraging the benefits provided by various pretreatments used in the production of chips. The findings indicate that a combination of citric acid and blanching pretreatments can be used to reduce the negative effects of vacuum-assisted infrared drying, which provides rapid drying, on the product and to produce more flavourful and appealing melon chips.

## INTRODUCTION

Melons, which have high economic value, are widely cultivated in warm and temperate climates. Due to their high nutritional value and pleasant taste and aroma, they are enjoyed and consumed worldwide. Tokuşoğlu (*1*) reported that the potassium, magnesium, calcium, β-carotene, total phenolic content (expressed as gallic acid equivalents), and antioxidant capacity of the Kırkağaç melon variety were 36 324 μg/g, 1309 μg/g, 1345 μg/g, 92.6 mg/kg, 233.4 mg/kg and 12.1 mg/mL, respectively. Although melons are generally consumed fresh, dried melon is the second most common way of consumption. Drying crops has long been a common practice to prolong their shelf-life.

However, drying causes negative changes in the texture and appearance of the product (shrinkage, cracking, hardening) and significant losses of nutrients, flavour and colour compounds (*2*). In recent years, innovative drying techniques such as fluid bed, lyophilisation, microwave, vacuum, infrared and spray drying have been developed to reduce adverse changes in products, as well as drying time and energy consumption (*3*). Compared to other innovative technologies, infrared technology stands out due to its fast processing, low cost and high energy efficiency. Therefore, it has become a frequently used technology in food drying processes in recent years (*4*). Melon slices have been dried by convective and infrared-assisted convective methods. While convective drying took 50 h, infrared-assisted convective drying was completed in 18 h (*5*). Infrared energy from the heat source is irradiated onto the heated surface. The energy density in the area reached by the rays is very high and this energy is absorbed directly by the inner layers of the material. Infrared waves do not require a medium to propagate; they can also propagate in a vacuum. This is one of the most important advantages of infrared radiation. The use of infrared radiation in combination with a vacuum is particularly successful in processing oxygen-sensitive products and prevents energy losses (*6*). Despite its many advantages, infrared drying has some disadvantages, such as crust formation, surface browning and partial burning. Drying at high infrared power, in particular, causes crust formation due to rapid heat transfer. Topuz *et al*. (*7*) and Uğurlu *et al*. (*8*) reported that the rehydration rates of dried pear and apple, respectively, decreased due to crust formation at high infrared power. Topuz *et al*. (*9*) found that the a* values and HMF contents of pear slices dried in a conventional dryer at 40, 55 and 70 °C were lower than those of samples dried in a vacuum in an infrared dryer at 100, 200 and 300 W (40, 55 and 70 °C).

Various pretreatments before drying are applied for purposes such as flavour formation, improving rehydration capacity, controlling shrinkage, enhancing appearance and colour, and preserving bioactive components in dried products. Different processes such as dipping, blanching, freezing and ultrasound have been used as pretreatments (*10*). Da Cunha *et al*. (*11*) evaluated the effects of ethanol, ultrasound, vacuum, and ultrasound-vacuum combination pretreatments on convectively dried melon. The pretreated samples dried faster than the control samples, and the losses in total phenolic content and ascorbic acid were lower. Tepe and Kadakal (*12*) reported that the shrinkage rate in melon samples dried convectively at 70 °C without pretreatment was 82.6 %, while the shrinkage rates of samples pretreated by blanching, 100 % ethanol, 0.5 % citric acid, and ultrasound ranged from 80.2 to 80.5 %. Studies of the effects of pretreatments on the quality characteristics of foods dried by infrared radiation are limited. In fact, we did not find any studies on multiple pretreatment applications in vacuum-assisted two-way infrared drying. In this study, the effects of multiple pretreatment combinations on drying time and product quality of melon chips dried by vacuum-assisted two-way infrared radiation are investigated.

## MATERIALS AND METHODS

### Sample preparation

Kırkağaç melon variety, obtained from a local market in Van province, Türkiye, was used in the study. The melons were quickly transferred to the laboratory and kept in a refrigerator at 4 °C until use. After the melons were washed and cleaned, the peel and seeds were removed. The flesh was cut into slices 4 cm wide, 5 cm long and 2–3 mm thick with a stainless-steel manual slicer. For all dipping pretreatments, 150 g of sample and 600 mL of solvent were placed in a 1000-mL glass beaker. The ratio of fruit slice to dipping solution (1:4, *m*/*V*) was determined by preliminary experiments. The following pretreatments were applied to the sliced melons.

Citric acid pretreatment (CA): melon slices were immersed in a 0.5 % citric acid (food grade, Balmumcu, Türkiye) solution prepared in water for 7 min at room temperature. These samples were considered the control group.

Citric acid/ethanol pretreatment (CA+ET): melon slices were immersed in 0.5 % citric acid solution prepared in 99.8 % ethanol (Carlo Erba, Val-de-Reuil, France) for 7 min.

Citric acid/ultrasound pretreatment (CA+US): melon slices were immersed in a 0.5 % citric acid solution prepared in water for 20 min. Ultrasound was applied at (30±2) °C and 100 % amplitude during immersion. The temperature was controlled with ice packs throughout all ultrasound procedures. The ultrasound-assisted immersion was performed using a Bandelin Sonoplus HD 3200 ultrasonic homogeniser (Berlin, Germany) equipped with Bandelin UW 3200 ultrasonic transducer (200 W max. power, 20 kHz) and VS70T titanium probe (*d*=13 mm).

Citric acid/ethanol/ultrasound pretreatment (CA+ET+US): melon slices were immersed in a 0.5 % citric acid solution prepared in 99.8 % ethanol for 20 min. Ultrasound was applied at 100 % amplitude during immersion.

Citric acid/blanching pretreatment (CA+BL): melon slices were immersed in a 0.5 % citric acid solution prepared in water at 80 °C for 7 min.

Citric acid/ethanol/ultrasound/blanching pretreatment (CA+ET+US+BL): melon slices were immersed in a 0.5 % citric acid solution prepared in 99.8 % ethanol at 78 °C for 20 min. Ultrasound was applied at 100 % amplitude during immersion.

Citric acid/freezing pretreatment (CA+FT): melon slices were frozen in a deep freezer (model D405DD; Şenocak, Manisa, Türkiye) at −24 °C for 16 h. Frozen melon slices were removed from the freezer and immediately thawed by immersion in a 0.5 % citric acid solution prepared in water at room temperature for 7 min.

Citric acid/ethanol/ultrasound/freezing pretreatment (CA+ET+US+FT): melon slices were frozen in a deep freezer at −24 °C for 16 h. Frozen melon slices were removed from the freezer and thawed by immersion in a 0.5 % citric acid solution prepared in 99.8 % ethanol for 20 min at room temperature. Ultrasound was applied at 100 % amplitude during immersion.

After pretreatment, excess water on the melon slices was removed using filter paper, followed by drying. Each pretreatment was applied in five replicates.

### Drying procedure

A vacuum-assisted two-way infrared oven (Uniterm, Ankara, Türkiye) was used for drying. The oven contained four 250 W infrared lamps, two on the ceiling and two on the floor. The temperature inside the oven was monitored with a thermocouple. The thermocouple probe was placed between the samples on the tray in a position where it was exposed to the light. The vacuum atmosphere inside the dryer was generated by a vacuum pump (model DOA-P730-BN; Pall Life Sciences, Benton Harbor, MI, USA). Melon slices were dried at 200 W infrared power (55 °C) and 60 kPa vacuum pressure until the final moisture of ˂9 %. A large perforated stainless-steel tray was used to receive more light from the infrared lamps on the floor of the oven. The distance between the samples and the lamps was 12 cm from both the top and the bottom. Approximately 150 g of pretreated fresh melon slices were placed on the tray for drying.

### Specific energy consumption

In the study, the total energy consumption required for the pretreatment and drying stages of 1 kg of melon was determined according to the following equation:


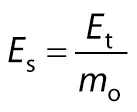
 /1/

where *E*_s_ is the total amount of specific energy (kWh/kg), *E*_t_ is the total amount of electrical energy (kWh) consumed during the processes, and *m*_0_ is the initial sample mass (kg). A power analyser (PM002+; WellHise, PR China) was used to measure the total electrical energy consumption (*13*).

### Moisture content

Moisture content was determined by drying the samples in weighed vessels at 105 °C until constant mass (*14*).

### Colour

The CIE colour values (L*, a* and b*) of the samples were determined using a PCE-CSM1 colourimeter (PCE Instruments, Meschede, Germany).

### Rehydration and shrinkage ratio

For the rehydration ratio, melon chips were placed in glass jars with a fruit/water ratio of 1:50 and rehydrated at 20 °C for 24 h. After rehydration, the chips were drained by placing them on a strainer for 30 s, the excess surface water was removed with a paper towel and the chips were weighed. The rehydration ratio was calculated according to the following equation (*15*):


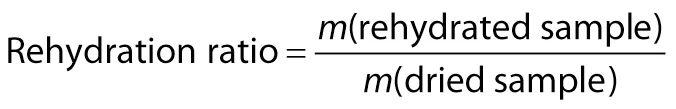
 /2/

The shrinkage ratio was analysed according to Reyes *et al*. (*16*) with some modifications. Fresh and dried samples (0.5 g) were placed in 5 mL of hexane. The volumes of fresh and dried samples were measured by the liquid displacement method and the shrinkage ratio was calculated according to the following equation:


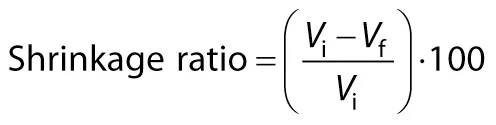
 /3/

where *V*_i_ is the initial volume (mL) and *V*_f_ is the final volume (mL).

### Sugar content

Sugars were extracted and analysed according to Míguez Bernárdez *et al*. (*17*). The extract was filtered through a 0.45-µm polyvinylidene difluoride (PVDF) syringe filter and injected into an HPLC system (LC-20 AD; Shimadzu, Kyoto, Japan) connected to a RID 20A refractive index detector. An Intersil NH2 column (4.6 mm×250 mm i.d., 5 µm; GL Sciences Inc., Tokyo, Japan) was used for separation at 25 °C. Acetonitrile/water (80:20) was used as the mobile phase in isocratic mode at a flow rate of 1.3 mL/min. Sugars on the chromatograms were identified by comparing retention times with those of standards. Results were expressed on dry mass basis as g/100 g. Total sugar content is given as the sum of individual sugars.

### HMF

HMF was extracted and analysed according to Alves *et al*. (*18*). The extracts were filtered through a 0.45-µm PVDF syringe filter and injected into the HPLC system (LC-20 AD; Shimadzu) connected to an SPD-M20A diode array detector. Waters Symmetry C18 column (250 mm×4.6 mm i.d., 5 µm; Waters, Wexford, Ireland) was used for separation at 25 °C. As the mobile phase, 2 % acetic acid in water (A) and 0.5 % acetic acid in water/acetonitrile (1:1) (B) were used with an elution profile of 10 % B at 0 min and 20 % B at 30 min. The flow rate was 1 mL/min. The HMF peak on the chromatogram was identified using the retention time and spectrum of a commercial standard (5-hydroxymethylfurfural; Carl Roth, Karlsruhe, Germany).

### Total phenolic content

Total phenolic content was determined according to Singleton and Rossi (*19*). A volume of 0.4 mL of sample extract, 2 mL of Folin-Ciocalteu reagent (10 % diluted with water) and 1.6 mL of sodium carbonate (7.5 %) were mixed. The mixture was kept in the dark for 60 min, after which the absorbance was measured at 765 nm. The results were expressed on a dry mass basis as mg gallic acid equivalents (GAE) per kg.

### Antioxidant activity (DPPH and ABTS)

The DPPH assay was performed according to Pyo *et al*. (*20*). A volume of 0.1 mL of extract was added to 3.9 mL of DPPH solution (0.025 g/L). The mixture was kept in the dark for 60 min, after which the absorbance was measured at 515 nm.

The ABTS assay was carried out according to Re *et al*. (*21*). Briefly, the ABTS^+^˙ was generated by reacting 7 mM ABTS with 2.45 mM potassium persulfate and incubating the mixture for 12–16 h at room temperature in the dark. The ABTS^+^˙ solution was then diluted with ethanol to an absorbance of 0.70±0.05 at 734 nm, after which 1980 μL of ABTS^+^˙ solution were mixed with 20 μL of extract. The mixture was incubated for 6 min at ambient temperature and its absorbance was measured at 734 nm. The results for DPPH and ABTS were expressed on dry mass basis as mmol Trolox equivalents (TE) per g.

### Sensory evaluation

Sensory analyses were carried out with 13 semi-trained and pre-informed panellists (6 females and 7 males, aged 25 to 40). Samples were randomly coded with three-digit numbers and presented to the panellists. The appearance, colour, odour, hardness, stickiness, taste, sourness and overall acceptability of dried melon chips were evaluated using a nine-point hedonic scale (*22*).

### Statistical analysis

Values were given as mean±standard deviation. For analysis of variance (one-way ANOVA) all data were analysed using IBM SPSS Statistics v. 25.0 (*23*). Duncan’s multiple comparison test was applied to evaluate the differences between the mean values. Correlation and principal component analysis (PCA) were carried out using SAS JMP v. 13.0 (*24*).

## RESULT AND DISCUSSION

### Processing times and specific energy consumption

The moisture content and processing times of melon chips are given in Table 1. The moisture content of fresh melon (89.4 %) was reduced to 6.6–9.0 % after drying. Tepe (*25*) found that the average moisture content of fresh melon was 89.6 % and approx. 10 % in dried melon. Our findings are similar to these results.

**Table 1:** Moisture content, pretreatment time, drying time and processing time of melon chips (*N*=5)

Sample	*w*(moisture)/%	*t*/min		
		Total pretreatment	Infrared drying	Total processing
Fresh	89.4±1.7	-	-	-
CA	(7.43±0.01)^cd^	7	155	162
CA+ET	(8.24±0.07)^ab^	7	135	142
CA+US	(6.6±0.2)^e^	20	135	155
CA+ET+US	(6.9±0.5)^ab^	20	92.5	112.5
CA+BL	(9.0±0.5)^a^	7	135	142
CA+ET+US+BL	(7.3±0.3)^cde^	20	90	110
CA+FT	(8.6±0.2)^de^	967	105	1072
CA+ET+US+FT	(8.0±0.3)^bc^	980	72.5	1052.5

Data are expressed as mean value±standard deviation. Lowercase letters in superscript indicate differences between samples (p<0.05). CA=citric acid, ET=ethanol, US=ultrasound, BL=blanching, FT=freezing

In the study, pretreatments CA, CA+ET, and CA+BL were shorter, while pretreatments CA+FT and CA+ET+US+FT, which included a freezing step, required longer times. Drying times of the samples varied between 70 and 155 min. Compared to the CA sample, the drying time decreased by 12.9–53.2 %. Blanching, ethanol, ultrasound, and freezing accelerated the drying process. In particular, CA+ET+US+FT and CA+ET+US+BL pretreatments had the shortest drying times at 72.5 and 90 min, respectively. Blanching, ultrasound and freezing increase the drying rate by damaging the cell wall, while ethanol increases the drying rate by dissolving the water in the tissue. CA+ET+US+FT and CA+ET+US+BL pretreatments caused both cell wall damage and the dewatering effect of ethanol. Therefore, they were more effective than individual treatments in reducing drying time. Otağ (*26*) found that different pretreatments caused differences in the drying rates of potato slices. When the samples were dried after immersion in 100 % ethanol solution, the drying rate was higher than that of the control sample.

The total specific energy consumption for pretreatment and drying processes in melon chip production varied between 2.6 and 4.7 kWh/kg (Fig. 1). All individual pretreatments reduced the total specific energy requirement compared to CA pretreatment as the positive control. Melon chips produced by freezing in particular had lower total specific energy consumption value than ethanol, ultrasound and blanching. The specific energy requirement for the drying process (2.2–4.7 kWh/kg) is higher than for pretreatment (0.02–0.83 kWh/kg). Compared to individual pretreatments, the combined pretreatments such as CA+ET+US, CA+ET+US+BL and CA+ET+US+FT significantly decreased the specific energy requirement by reducing drying time. The specific energy consumed for drying the samples using ethanol was greatly reduced. Similar results were reported by Kaveh *et al*. (*27*). Although CA+ET+US+FT pretreatment required a long processing time because of the freezing step, its specific energy requirement (2.6 kWh/kg), due to the shorter drying time, was the lowest of all pre-treatment applications. Motevali *et al*. (*28*) calculated the specific energy requirements for drying mushrooms using microwave, microwave-vacuum combination, infrared at different air velocities, and hot air-infrared combination as 2.5–6.0, 3.2–9.6, 47.9–93.5 and 15.0–71.1 kWh/kg, respectively. While reported results for microwave and microwave-vacuum drying are consistent with our findings, specific energy values for infrared drying at different air velocities and hot air-infrared combination are higher. This may be due to design differences. Two-way infrared drying combined with vacuum and pretreatment applications is a more suitable design to reduce specific energy requirements than the application of one-way infrared radiation and its combination with air velocity and hot-air drying.

**Fig. 1:**
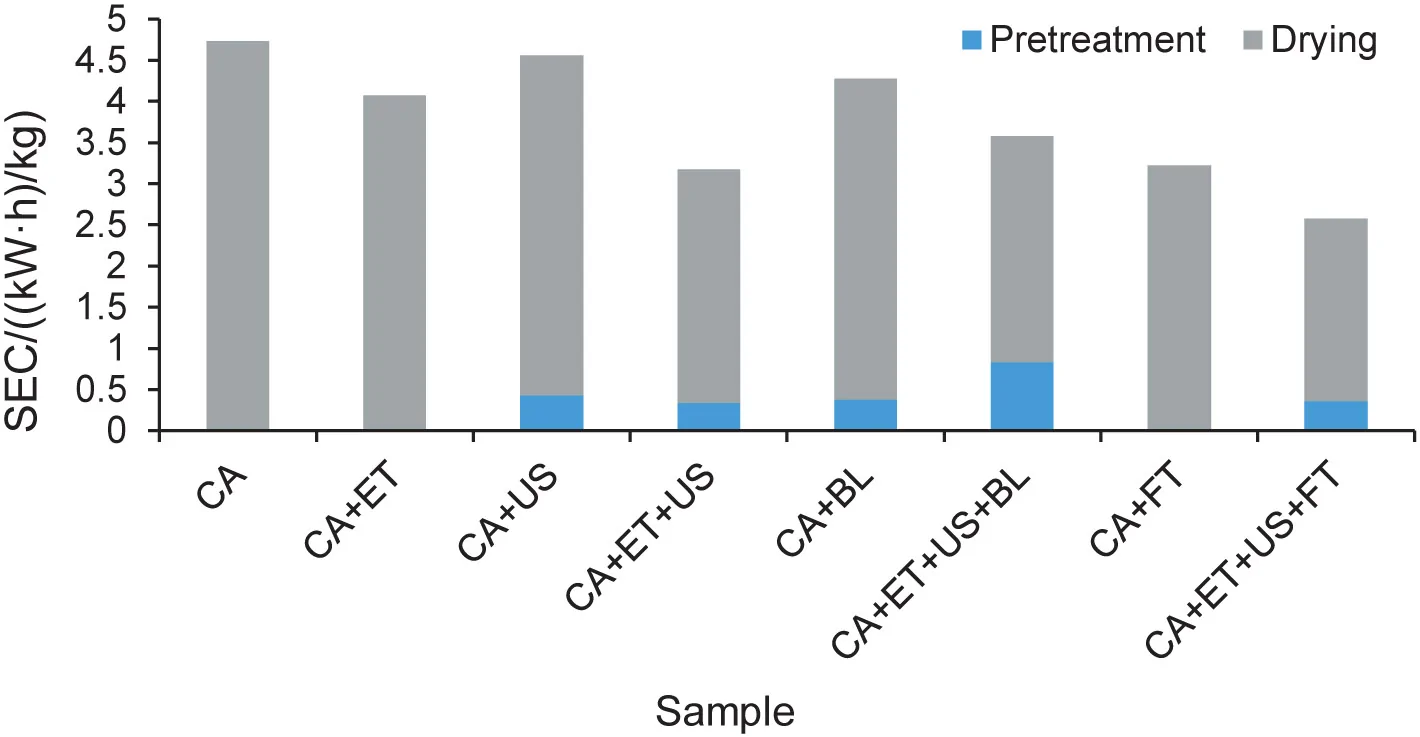
Specific energy consumption (SEC) in melon chips production. CA=citric acid, ET=ethanol, US=ultrasound, BL=blanching, FT=freezing

### Physical quality parameters of melon chips

The colour values of the samples are given in Table 2. The L*, a* and b* values of fresh melon were 59.2, 0.3 and 9.7, respectively. Da Cunha et al. (*11*) reported that L*, a* and b* values of fresh cantaloupe were 69.8, 18.2 and 38.6, respectively. Our results were higher than their findings. This may be associated with differences in ripening level, variety, geographical conditions and agricultural practices. The L*, a* and b* values of the melon chips were 56.7–74.2, 9.7–16.1 and 27.8–39.7, respectively. The drying process generally increased the L*, a* and b* values compared to fresh melon. Da Cunha et al. (*11*) reported that, after different pretreatments, the L*, a* and b* values of the dried cantaloupe ranged from 39 to 60, 17 to 24 and 31 to 40, respectively. The results obtained for dried cantaloupe are similar to the values in the present study. The differences among the samples produced with different pretreatments were statistically significant for L*, a* and b* values (p<0.05). The CA+US-treated sample had the lowest L* and the highest a* value. In particular, the samples produced with pretreatments including ultrasound application had lower L* and higher a* values than the untreated samples. Cell disruption and high temperature that occur during the ultrasound application may have caused a decrease in brightness and a darker colour. The CA+ET-treated sample exhibited the highest L* and lowest a* values among the melon chips. In terms of colour values, the samples immersed in ethanol showed the most favourable results. Da Cunha et al. (*11*) reported that the L* value decreased in dried melon samples pretreated with ultrasound. While the a* value decreased in samples pretreated with ethanol, their L* value was the highest. Rojas and Augusto (*29*) reported that ethanol pretreatment caused the dissolution and removal of pigments while dissolving cell wall compounds. As a result, the a* value decreased and brightness increased.

**Table 2:** Colour values, rehydration ratio, and shrinkage ratio of melon chips (*N*=5)

Sample	L*	a*	b*	Rehydration ratio/%	Shrinkage ratio/%
Fresh	59.2±5.9	0.3±0.7	9.6±3.0	-	-
CA	(65.8±4.8)^bc^	(11.2±0.9)^cd^	(31.6±2.5)^bc^	(3.6±0.2)^e^	(87.6±1.3)^cd^
CA+ET	(74.2±5.4)^a^	(9.7±0.5)^d^	(34.0±3.0)^b^	(5.5±0.1)^b^	(94.3±2.5)^a^
CA+US	(56.7±3.2)^d^	(16.1±1.2)^a^	(39.7±3.1)^a^	(4.2±0.2)^d^	(90.0±0.0)^bc^
CA+ET+US	(68.7±3.1)^ab^	(11.3±0.6)^cd^	(33.1±1.0)^bc^	(3.7±0.2)^e^	(92.0±0.0)^ab^
CA+BL	(60.4±1.5)^cd^	(11.7±0.9)^bc^	(30.8±1.2)^bcd^	(3.61±0.08)^e^	(91.6±0.6)^b^
CA+ET+US+BL	(60.1±3.7)^cd^	(13.3±1.2)^b^	(34.4±1.0)^b^	(5.00±0.05)^c^	(90.0±0.0)^bc^
CA+FT	(63.4±6.7)^bc^	(12.2±0.8)^bc^	(29.4±0.9)^cd^	(5.36±0.04)^bc^	(88.2±0.2)^cd^
CA+ET+US+FT	(67.2±1.1)^abc^	(9.8±1.3)^d^	(27.8±1.6)^d^	(6.1±0.4)^a^	(85.7±0.0)^d^

Data are expressed as mean value±standard deviation. Lowercase letters in superscript in the same column indicate differences between samples (p<0.05). CA=citric acid, ET=ethanol, US=ultrasound, BL=blanching, FT=freezing

The rehydration and shrinkage ratios of the dried samples in the vacuum-assisted two-way infrared dryer after different pretreatments were 3.6–6.1 and 85.7–94.3 %, respectively (Table 2). Tepe (*25*) reported that the rehydration and shrinkage rates of cantaloupe dried in a convective dryer after different pretreatments ranged from 5.3 to 5.9 and 80.1 to 80.6 %, respectively. While the values reported for rehydration were consistent with our results, the shrinkage values were slightly lower. This may be due to differences in melon varieties and drying methods used in the study. The effect of pretreatment combinations on the rehydration ratios of samples was significant (p<0.05). The highest rehydration ratio was observed after the CA+ET+US+FT pretreatment, while the lowest was found in the control (CA) sample. In the CA sample, there are no treatments affecting the cell wall. Ethanol, ultrasound and freezing pretreatments, which affect the cell wall, caused an increase in the rehydration ratio. Blanching was expected to have a similar effect, as it is a heat treatment. However, this effect was not fully observed in the CA+BL pretreatment, which may be due to the insufficient blanching time (7 min). Ethanol pretreatment (*30*) and freeze drying (*31*) have been reported to increase the rehydration capacity of the products.

The pretreatment combinations applied to the samples caused significant changes in the shrinkage rates (p<0.05). The highest shrinkage ratio was observed after CA+ET pretreatment, while the lowest was found after CA+ET+US+FT pretreatment. The shrinkage ratio of multiple pretreatment combinations such as CA+ET+US+BL and CA+ET+US+FT was lower than that of their single-process counterparts. CA+ET+US+FT had the highest drying rate, and the increase in drying rate may have reduced the shrinkage ratio. Aral and Beşe (*32*) reported that as the drying rate increases, mechanical stabilisation that limits shrinkage occurs on the surface of the food and this reduces the shrinkage ratio.

### Sugar and HMF contents of melon chips

Sugar content is an important quality indicator in melons and their products (*33*). The fructose, glucose, sucrose and total sugar mass fractions of fresh melon on a dry mass basis were 20.6, 22.4, 33.4 and 76.4 g/100 g, respectively, with sucrose as the major sugar (Table 3). Lignou *et al*. (*34*) reported that fructose, glucose and sucrose mass fractions in four different melon varieties on a dry mass basis varied from 20.8 to 21.9, 15.7 to 21.5 and 25 to 46 g/100 g, respectively. These values are consistent with those in our study. The sugar mass fraction of dried melon chips was lower than that of the fresh sample. The highest fructose, glucose and total sugar mass fractions in dry samples were found after CA and CA+FT pretreatments. The results show that ethanol, ultrasound and blanching, except for CA and freezing, lead to significant sugar loss. The differences between the pretreatments were statistically significant for fructose, sucrose and total sugar values (p<0.05). The greatest losses in total sugar content were observed after CA+BL pretreatment, which includes blanching. When comparing CA+BL and CA+ET+US+BL pretreatments, the higher losses after CA+BL may be due to differences in the solubility of sugars in water and ethanol. Tang *et al*. (*35*) reported that traditional blanching pretreatment causes significant losses in sugar content. Therefore, the temperature of the water used for blanching should be selected to cause as little damage as possible to the texture and sugar content of the product.

**Table 3:** HMF and sugar content of melon chips (*N*=5)

Sample	*w*(HMF)/(mg/kg)	*w(*sugar)/(g/100 g)			
		Fructose	Glucose	Sucrose	Total sugar
Fresh		20.6±1.4	22.4±3.2	33.4±3.9	76.4±5.2
CA	(41.9±1.1)^f^	(20.11±0.05)^a^	(20.9±0.6)^a^	(24.36±0.08)^bc^	(63.3±0.9)^a^
CA+ET	(60.1±1.6)^d^	(16.5±1.6)^a^	(17.4±2.6)^a^	(25.2±2.8)^bc^	(59.1±4.8)^ab^
CA+US	(118.0±2.8)^a^	(16.1±1.3)^a^	(17.1±0.6)^a^	(23.3±1.4)^bc^	(56.5±0.6)^b^
CA+ET+US	(74.9±0.8)^c^	(15.6±0.9)^a^	(15.2±1.8)^a^	(27.3±0.7)^a^	(58.0±0.8)^ab^
CA+BL	(52.8±0.1)^e^	(13.63±0.03)^ab^	(15.1±2.2)^a^	(17.9±0.4)^c^	(46.7±2.6)^b^
CA+ET+US+BL	(82.5±0.7)^b^	(15.52±0.05)^a^	(13.9±0.5)^a^	(28.5±0.2)^b^	(58.3±0.7)^ab^
CA+FT	(48.8±0.3)^e^	(18.8±1.0)^a^	(19.8±0.6)^a^	(25.1±2.6)^bc^	(63.7±3.0)^a^
CA+ET+US+FT	(80.9±0.7)^b^	(9.5±1.1)^b^	(17.3±1.1)^a^	(16.0±1.2)^c^	(42.8±0.9)^b^

Data are expressed as mean value±standard deviation. Lowercase letters in superscript in the same column indicate differences between samples (p<0.05). HMF=5-hydroxymethylfurfural, CA=citric acid, ET=ethanol, US=ultrasound, BL=blanching, FT=freezing

HMF is an indicator used to determine excessive heat treatment in thermal processes. The HMF mass fractions of chips on dry mass basis ranged between 41.9 and 118.0 mg/kg (Table 3). Tepe (*25*) found that HMF values in melon dried with different pretreatments and drying methods ranged from 0.1 to 8.9 mg/kg. These values are much lower than ours, which may be due to differences in melon varieties, drying methods, pretreatments and applied parameters. In addition, the high sugar mass fraction of the melon and the effect of thin slices may have contributed to the increase in HMF contents. The HMF mass fractions of CA, CA+ET, CA+BL and CA+FT samples was low. Pretreatments involving ultrasound (CA+US, CA+ET+US, CA+ET+US+BL and CA+ET+US+FT) led to higher HMF mass fraction. The effect of ultrasound on the increase in HMF was greater than that of ethanol, blanching and freezing. This may be due to tissue damage and local heat increase as a result of cavitation during ultrasound application. The highest HMF mass fraction was also found in the CA+US-treated sample (p<0.05). Although the CA+US pretreatment has a similar cavitation effect as the other pretreatments that include ultrasound, the drying time of the samples pretreated by CA+US is longer (p<0.05). The differences among the pretreatments in terms of HMF values were statistically significant (p<0.05).

### Bioactive compounds of melon chips

Total phenolic contents and antioxidant activities of melon chips are shown in Table 4. The total phenolic content, expressed as GAE on a dry mass basis, and the DPPH and ABTS assay results, expressed as TE on a dry mass basis, for fresh melon were 2785 mg/kg, and 17.3 and 22.2 mmol/g, respectively. These values for melon chips were 1702–2565 mg/kg and 4.9–7.5 and 5.2–14.9 mmol/g, respectively. The total phenolic content and antioxidant activity of melon chips were lower than those of fresh samples (p<0.05). Pretreatments and drying caused a loss of antioxidant compounds. A study by Kaveh *et al*. (*27*) reported similar results and showed losses of TPC and antioxidant activity in dried rose petals pretreated with ultrasound, ethanol and their combinations. The highest total phenolic content and antioxidant activity values were found after CA and CA+ET pretreatments. The total phenolic content and antioxidant activity values of samples prepared by multiple pretreatments were lower than those of the CA sample (control). The decreases observed in the CA+BL- and CA+FT-treated samples were similar. Blanching and freeze-thawing pretreatments may cause physical damage to cell structures and increase the porosity and surface area of the samples; they may accelerate the release of bioactive compounds during drying, followed by their degradation or oxidation (*36*). Losses were particularly high in multiple pretreatments that included ultrasound. The greatest loss of phenolic compounds and the lowest antioxidant activity values were observed in the CA+ET+US+FT-treated sample. Generally, antioxidant activity values and total phenolic content of samples changed in the same way. The differences in total phenolic content, DPPH and ABTS values between samples were statistically significant (p<0.05). Da Cunha *et al*. (*11*) reported that the total phenolic content of fresh cantaloupe, expressed as GAE on a dry mass basis, was 3.7 mg/g and that drying reduced the total phenolic content. Tepe (*25*) stated that while the DPPH value of fresh melon, expressed as TE on a dry mass basis, was 4.4 mmol/g, DPPH values of melon dried after different pretreatments ranged from 1.3 to 2.1 mmol/g. Nicoli *et al*. (*37*) reported that during food processing phenolic compounds undergo chemical, enzymatic or thermal degradation and consequently, antioxidant activity decreases.

**Table 4:** Total phenolic content, DPPH and ABTS values of melon chips (*N*=5)

Sample	*w*(total phenolic as GAE)/(mg/kg)	*b*(TE)/(mmol/g)	
		DPPH	ABTS
Fresh	2785±20	17.3±0.4	22.2±7.3
CA	(2565±40)^a^	(7.5±0.4)^a^	(13.4±1.4)^ab^
CA+ET	(2492±55)^a^	(7.1±0.4)^a^	(14.9±2.1)^a^
CA+US	(2035±102)^bc^	(7.0±04)^a^	(11.6±2.9)^abc^
CA+ET+US	(1992±142)^b^	(6.9±0.9)^a^	(8.7±2.5)^c^
CA+BL	(2097±354)^bc^	(7.0±0.2)^a^	(10.4±0.3)^bc^
CA+ET+US+BL	(1962±103)^bc^	(6.2±1.1)^ab^	(11.83±0.06)^abc^
CA+FT	(2134±34)^b^	(6.4±1.6)^a^	(10.5±1.2)^bc^
CA+ET+US+FT	(1702±61)^c^	(4.9±0.5)^b^	(5.2±1.0)^d^

Data are expressed as mean value±standard deviation. Lowercase letters in superscript in the same column indicate differences between samples (p<0.05). GAE=gallic acid equivalents, TE=Trolox equivalents, CA=citric acid, ET=ethanol, US=ultrasound, BL=blanching, FT=freezing

### Sensory attributes of melon chips

The results of the sensory evaluation are shown in Table 5. Significant differences were found among the samples except for odour and taste (p<0.05). The most liked samples in terms of appearance were those treated with CA+US, CA+ET+US and CA+ET+US+BL. The sample treated with CA+ET+US+BL had the highest colour score. The sample treated with CA+ET had the best colour values (L*, a* and b*), while the CA+ET-treated sample received the lowest colour score in the sensory evaluation. This may be due to the habit of people in Türkiye of consuming darker coloured sun-dried products. The CA+ET+US+FT- and CA+BL-treated samples were similar and had the highest scores for hardness and stickiness, while the CA+BL- and CA+ET-treated samples had the highest scores for sourness. The CA+BL-treated sample received the highest scores for several sensory parameters, especially for overall acceptability. This may be due to blanching in citric acid as it removes raw tastes and odours and has a positive effect on texture, with high hardness and stickiness scores. However, it was also determined that, compared to CA, multiple combinations did not cause dramatic declines in sensory scores. Multiple pretreatments, combining several positive effects, generally had higher sensory scores than CA.

**Table 5:** Sensory characteristics of melon chips

Sample	Appearance	Colour	Odour	Hardness	Taste	Stickiness	Sourness	Overall acceptance
CA CA+ET CA+US CA+ET+US CA+BL CA+ET+US+BL CA+FT CA+ET+US+FT	(7.1±1.5)^ab^ (5.5±2.6)^b^ (7.4±1.4)^a^ (7.4±1.4)^a^ (6.2±2.2)^ab^ (7.5±1.4)^a^ (6.4±1.6)^ab^ (6.7±2.1)^ab^	(7.0±1.7)^ab^ (4.9±2.5)^c^ (7.2±1.1)^ab^ (7.1±1.3)^ab^ (6.0±2.0)^bc^ (7.6±1.3)^a^ (6.1±1.7)^abc^ (6.6±2.0)^ab^	(6.3±2.0)^a^ (6.5±1.9)^a^ (6.1±1.5)^a^ (6.8±1.5)^a^ (6.4±1.9)^a^ (7.1±1.5)^a^ (7.2±1.5)^a^ (6.6±1.7)^a^	(4.6±1.7)^c^ (7.0±1.6)^a^ (5.5±2.1)^bc^ (6.3±2.0)^ab^ (7.6±1.3)^a^ (6.4±2.2)^ab^ (6.8±2.0)^ab^ (7.7±1.5)^a^	(5.8±2.1)^a^ (6.8±2.4)^a^ (5.8±2.1)^a^ (6.9±2.0)^a^ (7.2±1.4)^a^ (6.5±1.5)^a^ (6.6±2.3)^a^ (7.1±1.7)^a^	(6.0±2.3)^ab^ (6.7±2.1)^ab^ (5.8±2.1)^b^ (7.2±1.8)^ab^ (7.6±1.1)^a^ (6.1±2.6)^ab^ (6.6±2.2)^ab^ (7.8±1.5)^a^	(5.2±2.2)^b^ (7.3±1.3)^a^ (6.1±0.8)^ab^ (6.2±1.9)^ab^ (6.8±2.0)^a^ (6.1±2.0)^ab^ (6.5±1.9)^ab^ (6.5±1.6)^ab^	(6.3±2.2)^ab^ (5.8±2.2)^ab^ (5.6±1.9)^b^ (7.0±1.7)^ab^ (7.4±1.1)^a^ (6.2±1.9)^ab^ (6.3±2.0)^ab^ (7.0±1.4)^ab^

Data are expressed as mean value±standard deviation. Lowercase letters in superscript in the same column indicate differences between samples (p<0.05). CA=citric acid, ET=ethanol, US=ultrasound, BL=blanching, FT=freezing

### PCA analysis

The PCA results are presented as a biplot (Fig. 2). The first three principal components were accounted for 38.0, 23.3 and 13.6 % of the variation, respectively. The cumulative proportion of variation reached 74.9 % of the total variation. The traits contributing to the high variation in the first PCA component (PC1) were moisture, rehydration rate (RR), sucrose, hardness, taste, stickiness and overall acceptability. Traits contributing to the second PCA component (PC2) were L*, a*, fructose, HMF, TPC, DPPH, ABTS, appearance and colour. The third PCA component (PC3) was related to b*, shrinkage, glucose, total sugar, odour and sourness. While RR had the lowest effect on variation, shrinkage had the highest. The effects of the other traits on variation were similar. The effects of a*, HMF, colour and appearance in PC2 were negative, while those of L*, TPC, DPPH and ABTS were positive. As expected, a strong negative correlation was found between L* and a* values. This was a result of the enzymatic or non-enzymatic browning reactions that occur during pretreatment and drying. Although there was a strong positive correlation between TPC and antioxidant activity, no correlation among a*, HMF and antioxidant activity was found. The results suggest that phenolic compounds were the main contributors to the antioxidant activity of the melon chips, and browning pigments formed by Maillard reactions during pretreatment and drying had no or only limited effect on antioxidant activity. Significant correlations were also identified between taste and hardness, taste and stickiness, overall acceptability and stickiness, and taste and overall acceptability. They showed that the physical state of melon chips in the mouth had a greater effect on taste and overall acceptability than other sensory properties. Colour was the most influential parameter for appearance and therefore the highest correlation was found between colour and appearance. Samples produced using ultrasound had high HMF content, high a* values and dark colours. Since people in Türkiye commonly consume sun-dried dark-coloured products, the panellists gave high scores to samples with high HMF and a* values for colour and appearance. As a result, the a*, b*, HMF, colour and appearance parameters contributed most to the distribution models of the ultrasonic effect (below and to the right of the PC1 axis) (Fig. 2). CA was clearly positioned in the same quadrant (below and to the right of the PC1 axis) as sugars, TPC and antioxidant activity. CA was characterised by better preservation of sugars, phenolic content and antioxidant activity (ABTS and DPPH). A clear distinction was observed between CA+BL and CA treatments, and between CA+BL and treatments including ultrasound. CA+BL was located far from sugar, phenolic and antioxidant activity parameters, in the same region as sensory parameters except colour and appearance. Compared to other samples, the CA+BL-treated sample was characterised by high loss of sugars and moderate loss of TPC and antioxidant activity due to the effect of heat and extraction during blanching. However, it had the highest liking scores in sensory evaluation. While only the CA-treated sample, used as the control, was located in the positive region, CA+ET, CA+FT and CA+BL were clustered in the region opposite CA. All treatments involving ultrasound and multiple combinations were located in the negative ordinate region. In particular, the CA+ET+US+FT-treated sample stood out distinctly from the others.

**Fig. 2:**
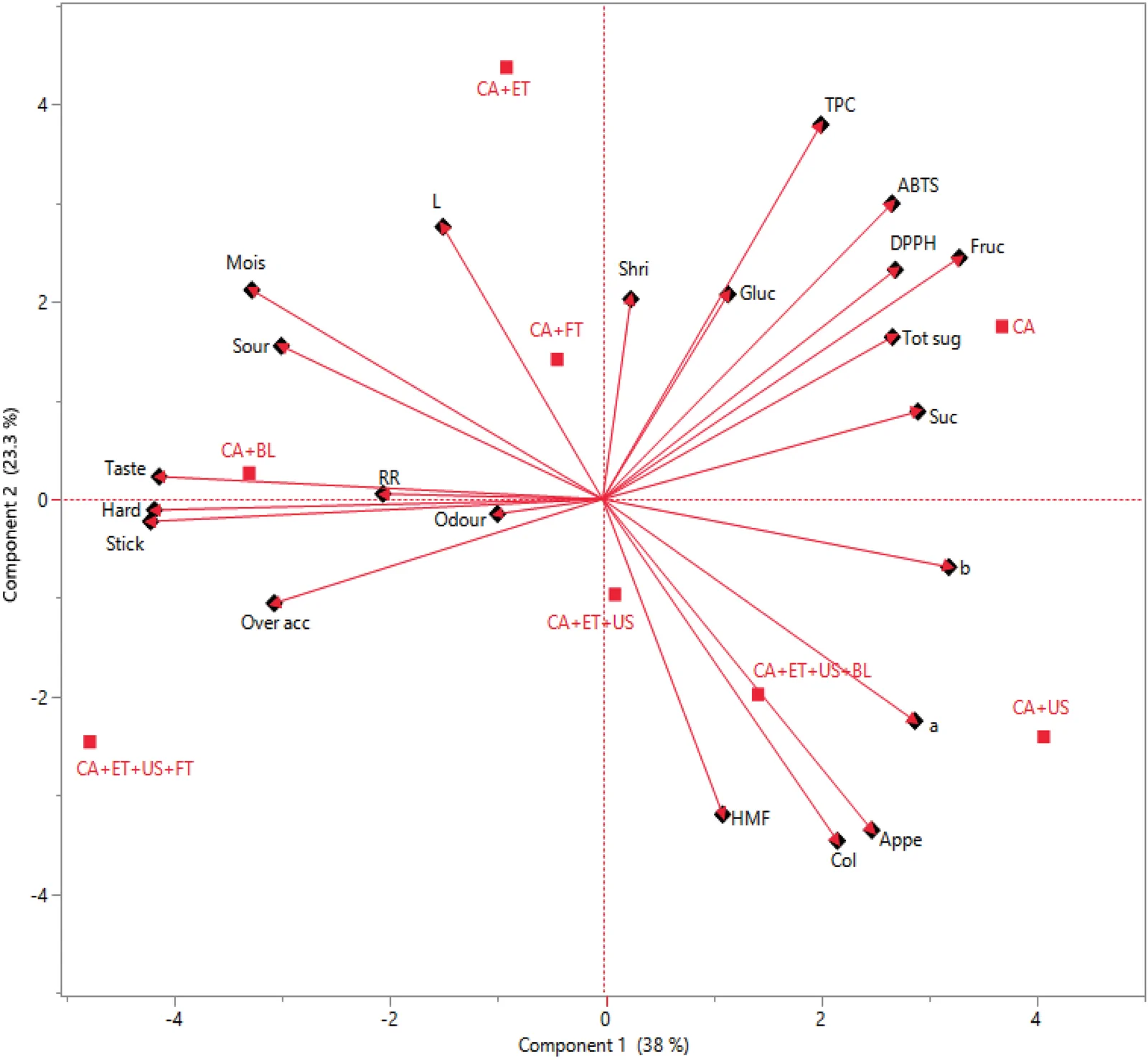
Biplot PCA results. Pretreatments: CA=citric acid, CA+ET=citric acid/ethanol, CA+US=citric acid/ultrasound, CA+ET+US=citric acid/ethanol/ultrasound, CA+BL=citric acid/blanching, CA+ET+US+BL=citric acid/ethanol/ultrasound/blanching, CA+FT=citric acid/freezing, CA+ET+US+FT=citric acid/ethanol/ultrasound/freezing. L=lightness, a=redness, b=yellowness, Mois=moisture, RR=rehydration rate, Shri=shrinkage ratio, TPC=total phenolic content, ABTS=ABTS scavenging activity, DPPH=DPPH scavenging activity, Gluc=glucose, Fruc=fructose, Suc=sucrose, Tot sug=total sugar, HMF=5-hydroxymethylfurfural, Col=colour, Odour=odour, Appe=appearance, Sour=sourness, Hard=hardness, Stick=stickiness, Over acc=overall acceptance

## CONCLUSIONS

Various pretreatments are used to eliminate or reduce many negative effects of drying on the product. As an innovative drying technique, vacuum-assisted two-way infrared drying has some disadvantages, such as high heat transfer rates and fast drying. This study investigated the effect of different pretreatment combinations on the quality characteristics of melon chips produced by vacuum-assisted two-way infrared drying. Among the different pretreatments (freezing, blanching, ultrasound, and immersion in ethanol and citric acid), freezing was the pretreatment that shortened the drying time the most. In particular, the use of freezing in combination with other pretreatments (immersion in citric acid and ethanol, ultrasound treatment and freezing) resulted in the shortest drying time and the lowest specific energy consumption. This approach improved the rehydration and shrinkage properties of melon chips. However, it significantly reduced the phenolic content and antioxidant activity of the samples. The application of ultrasound led to substantial increases in 5-hydroxymethylfurfural (HMF) content. The combination of citric acid and blanching was the most suitable pretreatment for the production of melon chips using two-way infrared drying under vacuum. Despite the high specific energy requirement and infrared drying time, this method offers short pretreatment time, low HMF content, moderate phenolic content and antioxidant activity, and the highest overall acceptance score. However, the absence of drying kinetics data is one of the limitations of this study. The results showed that many disadvantages (such as surface crusting and browning) caused by rapid drying in vacuum-assisted two-way infrared dryers can be eliminated by using various pretreatment combinations. This will enable the production of high-quality fruit chips with shorter processing times and lower energy consumption.
